# Effect of early metoprolol before PCI in ST‐segment elevation myocardial infarction on infarct size and left ventricular ejection fraction. A systematic review and meta‐analysis of clinical trials

**DOI:** 10.1002/clc.23894

**Published:** 2022-08-30

**Authors:** Karam R. Motawea, Hamed Gaber, Ravi B. Singh, Sarya Swed, Salem Elshenawy, Nesreen Elsayed Talat, Nawal Elgabrty, Sheikh Shoib, Engy A. Wahsh, Pensée Chébl, Sarraa M. Reyad, Samah S. Rozan, Hani Aiash

**Affiliations:** ^1^ Faculty of Medicine Alexandria University Alexandria Egypt; ^2^ Department of Internal Medicine Suny Upstate Medical university Syracuse New York USA; ^3^ Faculty of Medicine Aleppo University Aleppo Syria; ^4^ Department of Psychiatry Jawahar Lal Nehru Memorial Hospital Srinagar Jammu and Kashmir India; ^5^ Department of Clinical Pharmacy, Faculty of Pharmacy October 6 university Giza Egypt; ^6^ Department of Cardiovascular perfusion Upstate Medical University Syracuse New York USA

**Keywords:** metoprolol, PCI, ST‐segment elevation myocardial infarction

## Abstract

**Aim:**

This meta‐analysis aims to look at the impact of early intravenous Metoprolol in ST‐segment elevation myocardial infarction (STEMI) before percutaneous coronary intervention (PCI) on infarct size, as measured by cardio magnetic resonance (CMR) and left ventricular ejection fraction.

**Methods:**

We searched the following databases: PubMed, Scopus, Cochrane library, and Web of Science. We included only randomized control trials that reported the use of early intravenous Metoprolol in STEMI before PCI on infarct size, as measured by CMR and left ventricular ejection fraction. RevMan software 5.4 was used for performing the analysis.

**Results:**

Following a literature search, 340 publications were found. Finally, 18 studies were included for the systematic review, and 8 clinical trials were included in the meta‐analysis after the full‐text screening. At 6 months, the pooled effect revealed a statistically significant association between Metoprolol and increased left ventricular ejection fraction (LVEF) (%) compared to controls (mean difference [MD] = 3.57, [95% confidence interval [CI] = 2.22–4.92], *p* < .00001), as well as decreased infarcted myocardium(g) compared to controls (MD = −3.84, [95% [CI] = −5.75 to −1.93], *p* < .0001). At 1 week, the pooled effect revealed a statistically significant association between Metoprolol and increased LVEF (%) compared to controls (MD = 2.98, [95% CI = 1.26−4.69], *p* = .0007), as well as decreased infarcted myocardium(%) compared to controls (MD = −3.21, [95% CI = −5.24 to −1.18], *p* = .002).

**Conclusion:**

A significant decrease in myocardial infarction and increase in LVEF (%) was linked to receiving Metoprolol at 1 week and 6‐month follow‐up.

**Abbreviations**
CMR: cardio magnetic resonance
LVEDV: left ventricular end‐diastolic volume
LVEF%: left ventricular ejection fraction%
LVESV: left ventricular end‐systolic volume
MI: myocardial infarction
MIS: myocardial infarct size
PCI: percutaneous coronary intervention
Ppci: primary percutaneous coronary intervention
PRISMA: preferred reporting items for systematic reviews and meta‐analyses
ROB 2: risk of bias 2
STEMI: ST‐segment elevation myocardial infarction

## 1 INTRODUCTION

ST‐segment elevation myocardial infarction (STEMI) is the most important condition with a high mortality rate and hospitalization for coronary artery disease patients. It requires urgent and immediate intervention.^1^ A new era of enhancement in the outcome of STEMI patients has emerged.^2^ Although recurrent cardiovascular events such as congestive heart failure, arrhythmia, and sudden death are the significant probability incidence for STEMI survivors.^3^, ^4^

The standard reperfusion strategy is timely reperfusion with primary percutaneous coronary intervention (PPCI), ideally within 120 min of STEMI diagnosis.^5^, ^6^ Administration of beta‐blockers before PCI can decrease ischemic injury due to their impact on reducing myocardial contractility, slowing heart rate, lowering systemic blood pressure, and inhibiting neutrophils function, which in turn diminish reperfusion injury, especially with metoprolol.^7^

Current STEMI guidelines adopted early intravenous b‐blockade for the STEMI population at the time of presentation before PCI, ensuring no contraindications, no signs of acute heart failure, and the systolic blood pressure is >120 mmHg. In response to the (METOCARD‐CNIC) Trial that revealed the beneficial effect of Metoprolol in Cardioprotection through an acute myocardial infarction (MI).^8^, ^9^

Metoprolol significantly exhibits a decrease in myocardial infarct size and preserved left ventricular (LV) function, which was demonstrated by magnetic resonance imaging 1‐week postinfarction.^10^ In addition, long‐term left ventricular ejection fraction (LVEF) improvement, fewer indications for cardioverter‐defibrillator implantation, and a decrease in heart failure readmissions.^11^

This meta‐analysis aims to study the effect of early intravenous metoprolol in STEMI before PCI on infarct size assisted by cardio magnetic resonance (CMR) and LVEF at 1‐week and 6‐month follow‐up periods. Also, we aim to study its safety.

## 2 METHODS

### 2.1 Study design

We did a meta‐analysis intending to determine the efficacy of early intravenous metoprolol in STEMI before PCI on infarct size and LVEF using CMR and LVEF. We also intend to investigate its safety. The protocol of this study is registered on Prospero. The number of registration is CRD42022304100.

### 2.2 Search strategy

Relevant randomized control trials were located using the search keywords ([Metoprolol] OR [Metocard]) AND ([STEMI] AND [MI]) in PubMed, Scopus, Cochrane library, and Web of Science from inception to 18 January 2022.

### 2.3 Eligibility criteria

The following are the inclusion criteria for this meta‐analysis: (1) Randomized control clinical trials are one type of study, (2) STEMI is a type of patient. Patients with anterior STEMI, Killip class‐II, and a symptom onset‐to‐reperfusion period of 6 h, (3) Early IV metoprolol delivery to STEMI patients is one type of intervention. Patients before PCI (4) types of controls: Control group who did not receive metoprolol.

### 2.4 Exclusion criteria

We excluded cohort studies, case reports, editorials, and animal studies.

### 2.5 Study selection process

In the initial screening step, two independent researchers (H. G. and N. G.) examined the searched papers' titles and/or abstracts to identify potentially included publications. Second, potential reports will be requested to be recovered. Third, the retrieved reports' whole texts will be evaluated independently by the same researchers. A three‐step screening process will determine the study's ultimate inclusion. Any disputes amongst researchers will be handled through dialog during the screening process.

### 2.6 Data extraction and management

Two independent researchers will use a standardized, predefined, pilot‐tested excel form to extract the following information: the first author's name, year of publication, country, study design, sample size, participant details, treatment and control interventions, duration of intervention, outcome measures, results, and safety data. Additionally, data will be retrieved to quantify the risk of bias (RoB). Dissension will be used to identify and settle discrepancies; data extraction was done by two authors s as a preliminary step and see if they agree.

### 2.7 RoB and strength of evidence

RoB: ROB 2 tool will be used to assess the RoB.

### 2.8 Data synthesis

Data will be analyzed using RevMan software 5.4, and sensitivity analysis may be used. Results will be presented in a fixed model if no heterogeneity was detected and a random model if significant heterogeneity was detected.

## 3 RESULTS

### 3.1 Literature search

**Figure 1:**
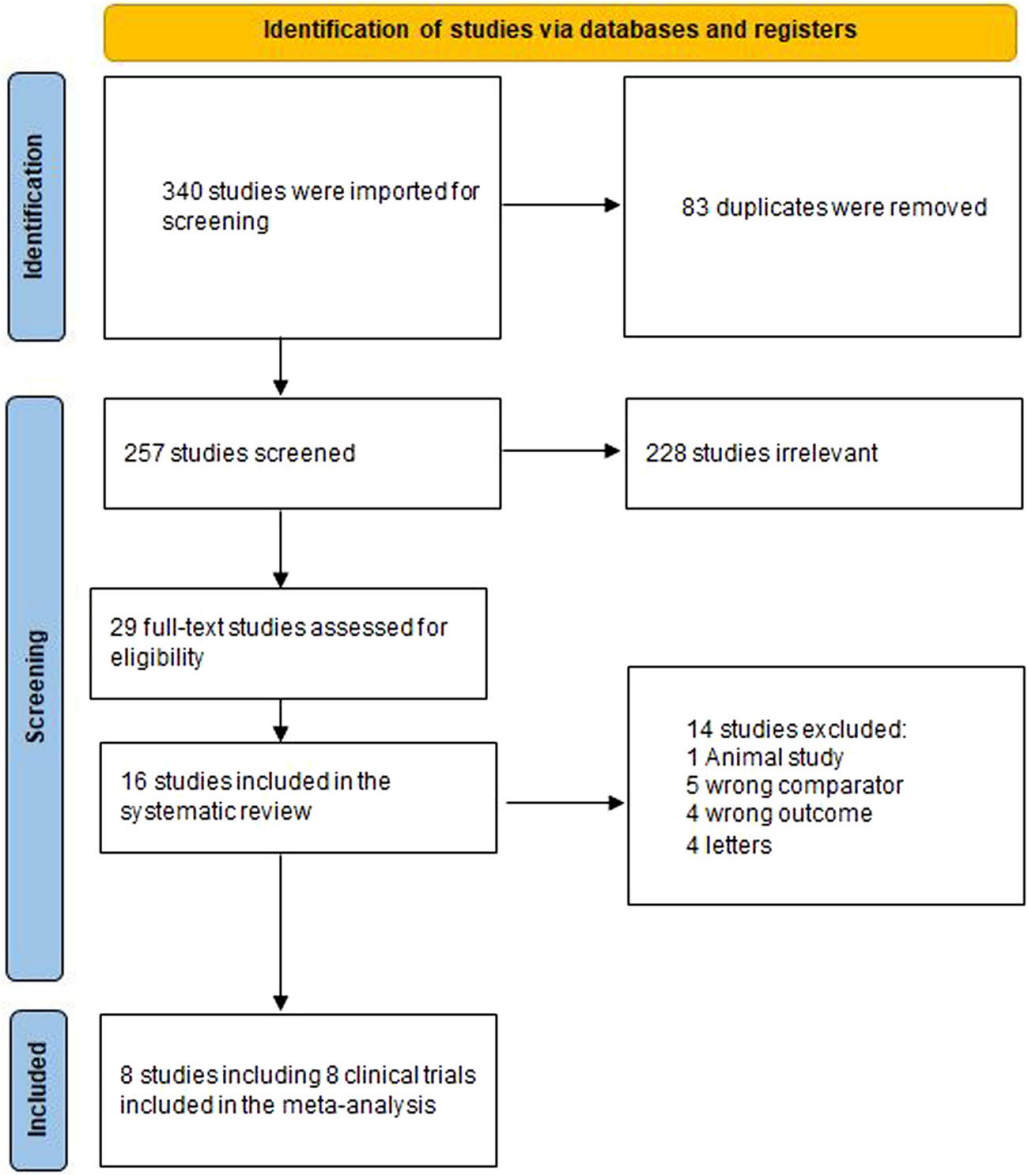
PRISMA flow diagram. PRISMA, preferred reporting items for systematic reviews and meta‐analyses

After a literature search, 340 publications resulted and then became 257 after removing duplicates. Of the 257, 228 were irrelevant, and 29 studies were eligible for full‐text screening. Finally, 16 studies were included for the systematic review, and eight clinical trials were included in the meta‐analysis after the full‐text screening, as shown in the preferred reporting items for systematic reviews and meta‐analyses (Figure 1). A summary of the studies is shown in Table 1.

**Table 1:** Summary of the included studies

ID	NCT	Trial region	Design	Duration	Study arms	Endpoints (outcomes)	Conclusion
**Pizarro 2014**	N/A	Spain	Multicenter randomized clinical trial	2 years	Patients with Killip‐class ≤II anterior STEMI presenting early after symptom onset (<6 h) and randomized them to prereperfusion i.v. metoprolol or control.	Mean (±*SD*) LV ejection fraction (LVEF) at 6 months magnetic resonance imaging (MRI) was higher after i.v. metoprolol (48.7 ± 9.9% vs. 45.0 ± 11.7% in controls; adjusted treatment effect 3.49%; 95% confidence interval [CI]: 0.44%–6.55%; *p* = .025). The occurrence of severely depressed LVEF (≤35%) at 6 months was significantly lower in patients treated with i.v. metoprolol (11% vs. 27%, *p* = .006). The proportion of patients fulfilling class‐I indications for implantable cardioverter‐defibrillator (ICD) was significantly lower in the i.v. metoprolol group (7% vs. 20%, *p* = .012). At a median follow‐up of 2 years, occurrence of the prespecified composite of death, heart failure admission, reinfarction, and malignant arrhythmia was 10.8% in i.v. metoprolol versus 18.3% in controls, adjusted hazard ratio (HR): 0.55; 95% CI: 0.26–1.04; *p* = .065. Heart failure admission was significantly lower in i.v. metoprolol (HR: 0.32; 95% CI: 0.015–0.95; *p* = .046). Conclusion: In patients with anterior Killip‐class ≤II STEMI undergoing pPCI, early i.v	In patients with anterior Killip‐class ≤II STEMI undergoing pPCI, early i.v. metoprolol before reperfusion resulted in higher long term LVEF, reduced incidence of severe LV systolic dysfunction and ICD indications, and fewer admissions due to heart failure.
**Podlesnikar 2020**	NCT01311700	Spain	Multicenter randomized clinical trial	6 months	Patients were randomized to receive intravenous metoprolol before reperfusion versus conventional therapy.	In the overall population, the infarct zone circumferential strain significantly improved from 1 week to 6 months after STEMI (−8.6 ± 9.0% to −14.5 ± 8.0%; *p* < .001), while no changes in the remote zone strain were observed (−19.5 ± 5.9% to −19.2 ± 3.9%; *p* = .466). Patients who received early intravenous metoprolol had significantly more preserved infarct zone circumferential strain compared to the controls at 1 week (*p* = .038) and at 6 months (*p* = .033) after STEMI, while no differences in remote zone strain were observed. The infarct zone circumferential strain was significantly impaired in patients with MVO and IMH compared to those without (*p* < .001 at 1 week and 6 months), however, it improved between both time points regardless of the presence of MVO or IMH (*p* < .001). In patients who developed adverse LV remodeling (defined as ≥20% increase in LV end‐diastolic volume) remote zone circumferential strain worsened between 1 week and 6 months after STEMI (*p* = .036), while in the absence of adverse LV remodeling no significant changes in remote zone strain were observed.	Regional LV circumferential strain with feature‐tracking CMR allowed comprehensive evaluation of the sequelae of an acute STEMI treated with primary percutaneous coronary intervention and demonstrated longlasting cardioprotective effects of early intravenous metoprolol.
**García‐Ruiz 2016**	N/A	Spain	A randomized clinical trial	6 months	Patients were randomized to receive long interval or short interval metoprolol or control before reperfusion.	For 218 patients (105 receiving IV metoprolol), the median time from 15 mg metoprolol bolus to reperfusion was 53 min. Compared with patients in the short‐interval group, those with longer metoprolol exposure had smaller infarcts (22.9 g vs. 28.1 g; *p* = .06) and higher left ventricular ejection fraction (LVEF) (48.3% vs. 43.9%; *p* = .019) on day 5 CMR. These differences occurred despite total ischemic time being signiﬁcantly longer in the long‐interval group (214 vs. 160 min; *p* < .001). There was no between‐group difference in the time from symptom onset to metoprolol bolus. In the animal study, the long‐interval group (IV metoprolol 25 min before reperfusion) had the smallest infarcts (day 7 CMR) and highest long‐term LVEF (day 45 CMR).	In anterior STEMI patients undergoing primary angioplasty, the sooner IV metoprolol is adminis‐tered in the course of infarction, the smaller the infarct and the higher the LVEF. These hypothesis‐generating clinical data are supported by a dedicated experimental large animal study.
**Tomaz Podlesnikar 2020**	NCT01311700	Spain, Netherlands	A multicenter, randomized, parallel‐group, single‐blinded (to outcome evaluators) clinical trial.	5 years	Patients with ﬁrst anterior STEMI enrolled in the randomized METOCARD‐CNIC clinical trial, assigned to receive intravenous metoprolol before primary percutaneous coronary intervention versus conventional STEMI therapy.	LV global circumferential (GCS) and longitudinal (GLS) strain were assessed with feature‐tracking CMR at 1 week after STEMI in 215 patients. The occurrence of major adverse cardiac events (MACE) at 5‐year follow‐up was the primary end point. Among 270 patients enrolled, 17 of 139 patients assigned to metoprolol arm and 31 of 131 patients assigned to control arm experienced MACE (HR: 0.500, 95% CI: 0.277–0.903; *p* = .022). Impaired LV GCS and GLS strain were signiﬁcantly associated with increased occurrence of MACE (GCS—HR: 1.208, 95% CI: 1.076–1.356, *p* = .001; GLS—HR: 1.362, 95% CI: 1.180–1.573, *p* < .001). On multivariable analysis, LV GLS provided incremental prog‐nostic value over late gadolinium enhancement (LGE) and LV ejection fraction (LVEF)(LGE + LVEF chi‐square = 12.865, LGE + LVEF + GLS chi‐square = 18.459; *p* = .012). Patients with GLS ≥−11.5% (above median value) who received early intravenous meto‐prolol were 64% less likely to experience MACE than their counterparts with same degree of GLS impairment (HR: 0.356, 95% CI: 0.129–0.979; *p* = .045).	Early intravenous metoprolol has a long‐term beneﬁcial prognostic effect, particularly in patients with severely impaired LV systolic function. LV GLS with feature‐tracking CMR early after percutaneous coronary intervention offers incremental prognostic value over conventional CMR parameters in risk stratiﬁcation of STEMI patients.
**Mateos 2014**	NCT01311700	Spain	A multicenter, randomized, parallel‐group, single‐blinded (to outcome evaluators) clinical trial.	6 months	For patients allocated to active treatment, metoprolol tartrate was administered intravenously and for control subjects did not receive any intravenous metoprolol before reperfusion, either from EMS or in the emergency department (ED).	From the total population of the trial (*N* = 270), 147 patients (54%) were recruited during out‐of‐hospital assistance and transferred to the primary angioplasty center (74 intravenous metoprolol and 73 controls). Infarct size was smaller in patients receiving intravenous metoprolol compared with controls (23.4 [*SD*: 15.0].	Out‐of‐hospital administration of intravenous metoprolol by EMS within 4.5 h of symptom onset in our subjects reduced infarct size and improved left ventricular ejection fraction with no excess of adverse events during the first 24 h.
**Ibanez 2012**	NCT01311700	Spain	A multicenter, randomized, parallel‐group, single‐blinded (to outcome evaluators) clinical trial.	1 week	Patients with Killip‐class‐II anterior ST‐segment elevation myocardial infarction (STEMI) undergoing PCI within 6 h of symptoms onset were randomized to receive i.v. metoprolol or not prereperfusion.	Patients with Killip class ≤II anterior ST‐segment elevation myocardial infarction (STEMI) undergoing PCI within 6 h of symptoms onset were randomized to receive i.v. metoprolol (*n* = 131) or not (control, *n* = 139) prereperfusion. All patients withoutcontraindications received oral metoprolol within 24 h. The predefined primary endpointwas infarct size on MRI performed 5–7 days after STEMI. MRI was performed in 220 patients (81%). Mean (±*SD*) infarct size by MRI was smaller after i.v.metoprolol compared to control (25.6 ± 15.3 vs. 32.0 ± 22.2 *g*; adjusted difference: −6.52; 95% CI: −11.39 to −1.78; *p* = .012). In patients with pre‐PCI TIMI flow grade0/1, the adjusted treatment difference in infarct size was −8.02; 95% CI: −13.01 to −3.02; *p* = .0029. Infarct size estimated by peak and area under the curve creatine kinase release wasmeasured in all study population and was significantly reduced by i.v. metoprolol. Left ventricular ejection fraction was higher in the i.v. metoprolol group (adjusted difference: 2.67%; 95% CI: 0.09%–5.21%; *p* = .045). The composite of death, malignant ventricular arrhythmia, cardiogenic shock, atrioventricular block and reinfarction at 24 h in the i.v. metoprolol and control groups respectively was 7.1% vs. 12.3%, *p* = .21.	In patients with anterior Killip‐class ≤II STEMI undergoing primary PCI, early i.v. metoprolol before reperfusion reduced infarct size and increased LVEF with no excess of adverse events during the first 24 h after STEMI.
**Podlesnikar 2018**	NCT01311700	Spain	Randomized clinical trial	6 months	patients with acute anterior STEMI who were allocated to intravenous metoprolol or control patients before primary PCI.	Patients who received early intravenous metoprolol had significantly more preserved LV strain compared with the control patients at 1 week after STEMI (GCS: −13.9 ± 3.8% vs. −12.6 ± 3.9%, respectively; *p* = .013; GLS: −11.9 ± 2.8% vs. −10.9 ± 3.2%, respectively; *p* = .032). In both groups, LV strain significantly improved during follow‐up (mean difference between 6‐month and 1‐week strain for the metoprolol group—GCS: −2.9%, 95% CI: −3.5% to −2.4%; GLS: −2.9%, 95% CI: −3.4% to −2.4%; both *p* < .001; the control group— GCS: −3.4%, 95% CI: −3.9% to −2.8%; GLS: −3.4%, 95% CI: −3.9% to −3.0%; both *p* < .001). When dividing the overall cohort of patients in quartiles of GCS and GLS, there were significantly fewer patients in the first quartile (i.e., the worst LV systolic function) who received early intravenous metoprolol compared with control patients at 1 week and 6 months (*p* < .05 for GCS and GLS at both time points).	In patients with anterior STEMI, early administration of intravenous metoprolol before primary PCI was associated with significantly fewer patients with severely depressed LV GCS and GLS, both at 1 week and 6 months. Feature‐tracking CMR represents a complementary tool to evaluate the benefits of cardioprotective therapies.
**Roolvink 2016**	NCT01569178	Netherlands	a multicenter, multinational, double‐blind, placebo‐controlled randomized clinical trial.	1 month	STEMI patients randomized to i.v. metoprolol or matched placebo before primary PCI.	No significant differences in baseline characteristics were observed. Infarct size (% of LV) by MRI did not differ between the metoprolol (15.3 ± 11.0%) and placebo group (14.9 ± *p* = .616). Peak and area under the creatine kinase (CK) curve did not differ between both groups. Left ventricular ejection fraction by MRI was 51.0 ± 10.9% in the metoprolol group and 51.6 ± 10.8% in the placebo group, *p* = .68. The incidence of malignant arrhythmias was 3.6% in the metoprolol group vs 6.9% in placebo *p* = .050. The incidence of adverse events was not different between groups. 11.5%	In a nonrestricted STEMI population, early intravenous metoprolol before pPCI, was not associated with a reduction in infarct size. Metoprolol reduced the incidence of malignant arrhythmias in the acute phase and was not associated with an increase in adverse events.
**Priti1 2017**	N/A	india	prospective double‐blind single‐center randomized controlled study	1 year	Of 1032 patients with acute inferior wall MI, 468 eligible patients were randomized in 1:1 manner to ivabradine (group A) and metoprolol (group B). Intention to treat analysis of 426 patients (group A‐232 and group B‐232) was performed.	Both the drugs decreased the mean heart rate to 62.22 ± 2.95 (group A) vs. 62.53 ± 3.59 (group B) beats per minute (*p* = .33). Ejection fraction improved in both the groups (5.15 ± 1.93% in group A vs 5.52 ± 2.18% in group B, *p* = .065). The two groups did not differ significantly in their primary endpoints in terms of death (group A = 1.72% vs. group B = 1.72%, odds ratio [OR] = 1.00, 95% CI = 0.25–4.05, *p* = 1.00), reinfarction (group A = 0.86% vs. group B = 0.86%, OR = 1.00, 95% CI = 0.14–7.16, *p* = 1.00), heart failure (group A = 4.31% vs. group B = 2.59%, OR = 1.70, 95% CI = 0.61–4.75, *p* = .31), or CHB (0% vs. 2.59%, OR = 0.07, 95% CI = 0.00–1.34, *p*= .08). There were no significant differences in the secondary endpoints of recurrent angina, readmission, and tachyarrhythmias except for more first‐and second‐degree AV blocks with metoprolol (12.93% vs. 2.59%, OR = 5.59, 95% CI = 2.28–13.72, *p* = .0002).	Ivabradine is well tolerated and equally effective as metoprolol in acute inferior wall ST elevation myocardial infarction patients for lowering the heart rate with lesser risk of AV blocks.
**Tölg 2006**	N/A	Germany	prospective, randomized, clinical study.	29 months	All other suitable patients who gave informed consent were 1:1 randomized to immediately receive either 50 mg metoprolol tartrate or 12.5 mg carvedilol per OS	Patients included in the study did not differ in age, gender and cardiovascular risk factors, such as arterialhypertension, diabetes, smoking habit, obesity, hyperlipidemia, hyperuricemia or family history ofcoronary heart disease (see Table 1). Hyperlipidemia was defined as known elevation of cholesterol without treatment, when specific medical treatment was already present irrespectively of actual lipid statusor elevation of cholesterol above accepted range for patients with coronary heart disease demonstrated within the first 3 days after hospital admission.	The present study demonstrates, for the first time in humans, that the administration of carvedilol before reperfusion with direct PCI in acute MI appears not to be superior to metoprolol in the limitation of myocardial injury and improvement of global and regional LV function in our small collective of patients. Our observations do not support expectations derived from experimental findings, which suggested that a particular benefit of carvedilol in experimental ischemia/reperfusion injury translates into clinical benefits in patients with acute myocardial infarction.
**Edwards 2008**	N/A	canada	Registry design and data collection (multicenter, multinational, prospective study)	9 years	The COMMIT/CCS‐2 trial randomized 45 852 patients with suspected ACS and ST‐segment deviation or left bundle branch block to receive metoprolol or placebo. Patients in the metoprolol arm received up to 15 mg of IV metoprolol in 3 divided doses at 2‐ to 3‐min intervals, followed by 200 mg of metoprolol daily.	Of the 14 231 patients with ACS, 77.7% received BB therapy within 24 h of presentation (78.5% and 77.4% in the STEMI and NSTEACS groups, respectively). The early use of BB declined in the STEMI group (80.3%–76.7%, *p* = .005) but increased in the NSTEACS group (75.4%–78.9%, *p* = .001) after 2005. Long‐term BB use, higher systolic blood pressure, and higher heart rate were independent predictors of early BB use. Conversely, patients who were female, older, Killip class N1, and had cardiac arrest at presentation were less likely to receive early BB. Multivariable analysis showed a trend toward lower use of BB among patients with STEMI (adjusted OR: 0.76, 95% CI: 0.57–1.00, *p* = .055) and a trend toward more frequent BB use among patients with NSTEACS (adjusted OR: 1.22, 95% CI: 0.96–1.55, *p* = .11) after 2005. The temporal trends in the early use of BB differed between patients with STEMI and patients with NSTEACS (*p* for interaction with period *b* = .001).	Most patients with STEMI or NSTEACS were treated with early BB therapy. In accordance with the COMMMIT/CCS‐2 trial, patients with lower systolic blood pressure and higher Killip class in the “real world” less frequently received early BB therapy. Since the publication of COMMIT/CCS‐2, there has been no significant change in the use of BB in patients with STEMI or NSTEACS after controlling for their clinical characteristics.
**FASULLO 2009**	N/A	Italy	Randomized clinical trial	2 years	Of the 567 consecutive patients admitted with acute myocardial infarction, only 155 patients (50 female, 105 males) with anterior STEMI met the entry criteria and were included into the study. These patients were randomized (double blind) in 2 groups: a group received b‐blockers (76 patients) 12 h after successful PCI and the other group received ivabradine (79 patients) 12 h after successful PCI.	Patients with a first anterior STEMI, Killip class‐I–II, an acceptable echocardiographic window, and admitted within 4 h of the onset of symptoms, with an ejection fraction 50%. METO or IVA, 12 h after PCI (double blind), were administered twice per day. Blood pressure (BP), heart rate (HR), electrocardiogram (ECG), and laboratory parameters were monitored during the study. At entry, days 10, 30, and 60, by echocardiography, the ESV, EDV, E/A ratio, E wave decelerationtime, isovolumetric relaxation time were measured. A total of 155 (50 females, 105 males) patients were randomized in 2 groups: a group received METO (76 patients) 12 h after PCI and a group received IVA (79 patients) 12 h after PCI. The 2 groups were similar for clinical characteristics. No difference was evidenced in HR, systolic blood pressure, diastolic blood pressure, age (range: 39–73 years), sex, ejection fraction (EF), creatine kinase peak, between the 2 groups at entry. Both groups were similar for time to angiography and interventional procedures and number of vessels diseased. IVA group: the 79 patients showed 2 side effects and 5 readmissions: 4 for ischemic events and 1 for heart failure, and 1 sudden death; METO group: the 76 patients had 4 ischemic events, 2 side effects, and 1 patient died during reeacute MI (intrastent thrombosis) and 8 readmissions for heart failure signs. The systolic blood pressure and diastolic blood pressure showed a significant reduction in both groups, *p* = .0001, respectively), and significant lower values were observed in METO group, *p* = .0001). The HR was significantly reduced in both groups, *p* = .0001). IVA group showed a significant increase in EF, *p* = .0001, with concomitant reduction in ESV and EDV (*p* = .0001, and .048, respectively). The diastolic parameters were similar in both groups during study period.	Our results suggest that ivabradine may be administered early (12 h after PCI) to patients with successful PCI for anterior STEMI with an impaired left ventricular function and high HR and sinus rhythm. A larger sample of patients and further studies are required to evaluate the effects of ivabradine on clinical endpoints.
**Chen 2005**	NCT 00222573.	China	randomized placebo controlled trial	5 years and 6 months	45 852 patients admitted to 1250 hospitals within 24 h of suspected acute MI onset were randomly allocated metoprolol (up to 15 mg intravenous then 200 mg oral daily; *n* = 22 929) or matching placebo (*n* = 22 923). 93% had ST‐segment elevation or bundle branch block, and 7% had ST‐segment depression. Treatment was to continue until discharge or up to 4 weeks in hospital (mean 15 days in survivors) and 89% completed it. The two prespecified coprimary outcomes were: (1) composite of death, reinfarction, or cardiac arrest; and (2) death from any cause during the scheduled treatment period. Comparisons were by intention to treat, and used the log‐rank method.	Neither of the coprimary outcomes was significantly reduced by allocation to metoprolol. For death, reinfarction, or cardiac arrest, 2166 (9.4%) patients allocated metoprolol had at least one such event compared with 2261 (9.9%) allocated placebo (OR: 0.96, 95% CI: 0.90–1.01; *p* = .1). For death alone, there were 1774 (7.7%) deaths in the metoprolol group versus 1797 (7.8%) in the placebo group (OR: 0.99, 95% CI: 0.92–1.05; *p* = .69). Allocation to metoprolol was associated with five fewer people having reinfarction (464 [2.0%] metoprolol vs 568 [2.5%] placebo; OR: 0.82, 95% CI: 0.72–0.92; *p* = .001) and five fewer having ventricular fibrillation (581 [2.5%] vs. 698 [3.0%]; OR: 0.83, 95% CI: 0.75–0.93; *p* = .001) per 1000 treated. Overall, these reductions were counterbalanced by 11 more per 1000 developing cardiogenic shock (1141 [5.0%] vs. 885 [3.9%]; ORL 1.30, 95% CI: 1.19–1.41; *p* = .00001). This excess of cardiogenic shock was mainly during days 0–1 after admission, whereas the reductions in reinfarction and ventricular fibrillation emerged more gradually. Consequently, the overall effect on death, reinfarction, arrest, or shock was significantly adverse during days 0–1 and significantly beneficial thereafter. There was substantial net hazard in haemodynamically unstable patients, and moderate net benefit in those who were relatively stable (particularly after days 0–1).	The use of early‐blocker therapy in acute MI reduces the risks of reinfarction and ventricular fibrillation, but increases the risk of cardiogenic shock, especially during the first day or so after admission. Consequently, it might generally be prudent to consider starting ‐blocker therapy in hospital only when the haemodynamic condition after MI has stabilized.
**Hjalmarson 1981**	N/A	Sweden	Randomized prospective studies	3 years and 5 months	1395 patients were included into the study and randomized to either metoprolol or placebo. Metoprolol was given 15 mg i.v. (5 mg every other minute), and 15 min later an oral dose of 50 mg was given which was repeated every 6 h for 48 h. From the third day metoprolol was given 100 mg twice daily.	Analysis of serum enzyme estimations of maximal LD I ± II showed a significant reduction by metoprolol when the treatment was given within 12 h of onset of pain. In a subgroup consisting of 103 pts. with AM1 metoprolol had no clearcut effects on the ventricular arrhyrmias during the first 24 h in hospital. The betablockade resulted in a 15% reduction in heart rare. The main objective of this study, the mortality during 3 months of blind treatment will be published late in 1981.	It can be summarized that a number of beneficial clinical effects of the betal‐blocking agents practolol and metoprolol have been demonstrated in patients with acute myocardial infarction. It is possible to reduce heart work and thereby myocardial metabolic demand causing an improvement of the ischemic myocardial metabolism. The reduction in myocardial ischemia would cause pain relief, a fall in the elevated ST‐segment and hopefully also, an improvement of left ventricular pump function. It seems possible also in man, to prevent and limit infarct development with metoprolol in agreement with similar suggestions from other studies utilizing propranolol, alprenolol and atenolol. There seems to be little or no effect of metoprolol on ventricular ectopic activity. The main objective of the double‐blind study of metoprolo1 in Goteborg, the mortality during the 3 months of blind treatment, is now under final analysis and data will be available during the early fall 1981.
**Rrannevik 1989**	N/A	N/A	Randomized clinical trial	2 weeks	Of 668 consecutive patients evaluated, 197 were randomized to metoprolol or placebo treatment (Figure 1). The 471 exclusions were due to current beta‐blocker treatment (*n* = 232; 49%) calcium channel blocker treatment (*n* = 70; 15%) bradycardia (*n* = 81; 17%) hypotension (*n* = 14; 3%), congestive heart failure (*n* = 20; 4%) obstructive lung disease (*n* = 12; 9%) and other and administrative reasons (*n* = 42; 9%). Within 15 days, 7 patients died (all cardiac deaths) and 9 patients had nonfatal reinfarctions.	One‐hundred and thirty‐two patients performed the early exercise test; 70 patients were treated with metoprolol and 62 with placebo. Several characteristics were compared in the two treatment groups. No demographic differences were observed. Supraventricular tachyarrhythmias occurred more frequently in the acute phase in the placebo‐treated patients (18 patients vs. 3, *p* = .0003).	Early administration of metoprolol to selected patients with suspected acute myocardial infarction is safe and probably limits infarct size [9.19‐221]. Exercise‐induced ST‐segment elevation and ventricular arrhythmias, but not ST‐segment depressions, are less frequently detected in these patients on predischarge exercise testing.
**Díaz‐Munoz 2021**	N/A	spain	METOCARD‐CNIC trial	6 months	The METOCARDCNIC trial randomized 270 anterior STEMI patients to IV metoprolol or control before reperfusion by percutaneous coronary intervention (PCI). In 139 patients (69 IV metoprolol, 70 controls), two ECGs were available (ECG‐1 before randomization, ECG‐2 pre‐PCI).	Between‐group ECG differences were analyzed using univariate and multivariate regression models. No significant between‐group differences were observed on ECG‐1. On ECG‐2, patients who received IV metoprolol had a narrower QRS than those in the control group (84 vs. 90 ms, *p* = .029), a lower prevalence of QRS distortion (10% vs. 26%, *p* = .017), and a lower sum of anterior and total ST‐segment elevation (10.1 vs. 13.6 mm, *p*=.014 and 10.4 vs. 14.0 mm, *p* = .015, respectively). Adjusted analysis revealed similar results. Significant associations were observed between ECG‐2 variables and cardiac magnetic resonance imaging measurements (extent of myocardial edema, infarct size, microvascular obstruction, and left‐ventricular ejection fraction) after STEMI.	In summary, IV metoprolol administration before reperfusion ameliorates ECG markers of myocardial ischemia in anterior STEMI patients. These data confirm that IV metoprolol is able to reduce ischemic injury and highlight the ability of ECG analysis to provide relevant real‐time information on the effect of cardioprotective therapies before reperfusion.

### 3.2 Characteristics

**Figure 2:**
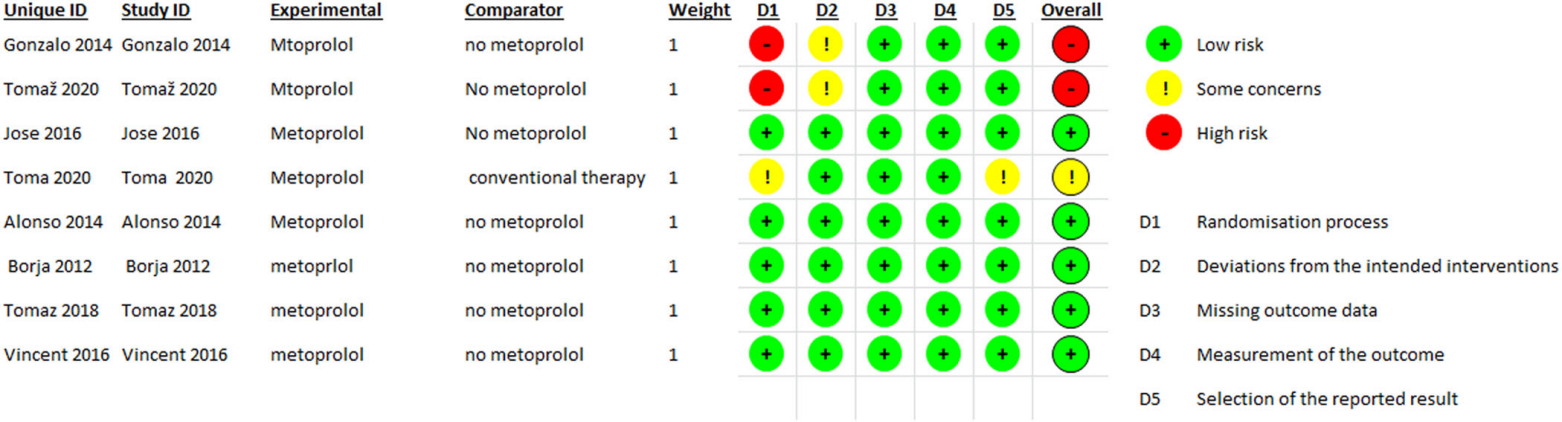
Risk of bias assessment

The results were reported on two follow‐up periods, 1 week and 6 months. Left ventricular end‐diastolic volume (LVEDV), Left ventricular end‐systolic volume (LVESV), left ventricular mass (LV mass), infarcted myocardium (%), and LVEF (%) as 1‐week follow‐up outcomes were reported in 3, 3, 3, 2, and 3 studies, respectively. LVEDV, LVESV, left ventricular mass (LV mass), Infarcted myocardium (g), Infarcted myocardium (%), and LVEF (%) as 6‐month follow‐up outcomes were reported in 6, 6, 4, 4, 5, and 6 studies, respectively. Major adverse cardiac events (MACE), death, heart failure admission, reinfarction, and malignant ventricular arrhythmia adverse events were all reported in 5, 4, 2, 5, and 4 studies, respectively. The adverse events were pooled at 6 months; only one study reported adverse events at 60 months. The overall RoB was low in 6 studies and high in 2 of the included studies, as shown in Figure 2.

The total number of patients included in the study is 1888 patients, 936 patients in the Metoprolol group, 952 patients in the control group, and other baseline data are shown in Table 2.

**Table 2:** Baseline characteristics of the included studies

ID	Study arms		Number of patients in each group		Age (Years)		sex (n)				other diseases N(%)	
	Intervention (the route of administration)	Control (Placeob or no control)	Intervention	Control	Intervention	Control	Intervention		Control			
							female	male	female	Male	Intervention	Control
**Pizarro 2014**	IV metoprolol	No intervention	101	101	49 ± 10.3	49 ± 10.3	_	_	_	_	_	_
**Podlesnikar 2020**	IV metoprolol	conventional therapy	97	94	57.4 ± 12.2	58.4 ± 10.2	14	83	9	85	Diabetes mellitus 21 (22) Previous hypertension 35 (36) Dyslipidemia 42 (43)	Diabetes mellitus 18 (19) Previous hypertension 37 (39) Dyslipidemia 41 (44)
**García‐Ruiz 2016**	IV metoprolol	No intervention	long interval=52 short interval= 53	113	Long interval (58.1 ± 12.8) short interval (59.0 ± 12.5)	(58.6 ± 10.4)	Long interval=8 short interval=6	Long interval=44 short interval=47	14	99	Diabetes mellitus Long I 11 (21.6) Short I 11 (20.8) Previous hypertension Long I 19 (37.3) Short I 20 (37.7) Dyslipidemia Long I 26 (51.0) Short I 18 (34.0)	Diabetes mellitus 21 (18.6) Previous hypertension 47 (41.6) Dyslipidemia 46 (40.7)
**Tomaz Podlesnikar 2020**	IV metoprolol	conventional therapy	139	131	Overall: 58.4 ± 11.5		Overall: Male 187 Female 28				Overall: Diabetes mellitus 42 (20) Previous Hypertension 84 (39)	
**Mateos 2014**	IV metoprolol	No intervention	74	73	59 ± 12	59 ± 10	12	62	13	60	Diabetes mellitus 12 (16.9) Previous hypertension 27 (39.1) Dyslipidemia 30 (44.1)	Diabetes mellitus 13 (17.8) Previous hypertension 30 (41.7) Dyslipidemia 27 (38)
**Ibanez 2012**	IV metoprolol	No intervention	139	131	58.7 ± 12.7	58.2 ± 10.8	20	119	17	114	Diabetes mellitus 31 (23.3) Previous hypertension 54 (40.3) Dyslipidemia 53 (39.8)	Diabetes mellitus 24 (18.8) Previous hypertension 54 (42.2) Dyslipidemia 51 (40.2)
**Podlesnikar 2018**	IV metoprolol	conventional therapy	100	97	57.8 ± 12.3	58.4 ± 10.1	13	87	11	86	Diabetes mellitus 21 (21) Previous hypertension 37 (37) Dyslipidemia 43 (43)	Diabetes mellitus 18 (19) Previous hypertension 37 (38) Dyslipidemia 42 (43)
**Roolvink 2016**	IV metoprolol	Placebo	336	347	62.39 ± 12.42	62.46 ± 12.58	84	252	88	259	Diabetes mellitus 48/335 Previous hypertension 135/335	Diabetes 62/347 Previous hypertension 133/344

**ID**	**Duration of disease (hours)**		**Killip class at recruitement N(%)**		**SBP, DBP, HR at inclusion (Mean** ± **SD)**	
	**Intervention**	**Control**	**Intervention**	**Control**	**Intervention**	**Control**
**Pizarro 2014**	<6 hours	<6 hours	class ≤II STEMI	class ≤II STEMI	_	_
**Podlesnikar 2020**	_	_	Class II 8 (8%)	Class II 11 (12%)	SBP 142 ± 18 DBP 90 ± 16 HR 82 ± 14	SBP 143 ± 20 DBP 87 ± 16 HR 81 ± 13
**García‐Ruiz 2016**	<6 hours	<6 hours	Class I Long I 48 (92.3%) Short I 48 (90.6%)	Class I 100 (88.5%)	SBP Long I 144 ± 20 Short I 141 ± 16 DBP Long I 93 ± 15 Short I 86 ± 17 HR Long I 80 ± 12 Short I 84 ± 15	SBP 142 ± 19 DBP 87 ± 15 HR 82 ± 14
**Tomaz Podlesnikar 2020**	_	_	_	_	_	_
**Mateos 2014**	≤4.5 hours	≤4.5 hours	Class I 67 (90.5%) Class II 7 (9.5%)	Class I 60 (82.2%) Class II 13 (17.8%)	SBP 143 ± 20 DBP 92 ± 15 HR 81 ± 11	SBP 142 ± 20 DBP 90 ± 14 HR 81 ± 14
**Ibanez 2012**	≤4.5 hours	≤4.5 hours	Class I 128 (92.1%) Class II 11 (7.9%)	Class I 114 (87%) Class II 17 (13%)	SBP 143 ± 19 HR 82 ± 14	SBP 142 ± 19 HR 82 ± 14
**Podlesnikar 2018**	_	_	Class II 8 (8%)	Class II 11 (11%)	SBP 142 ± 18 DBP 89 ± 16 HR 82 ± 14	SBP 142 ± 19 DBP 87 ± 15 HR 81 ± 13
**Roolvink 2016**	<12 hours	<12 hours	Killip I‐II	Killip I‐II	SBP 136.4 ± 22.91 DBP 82.25 ± 14.67 HR 74.35 ± 13.71	SBP 138.7 ± 26.43 DBP 82.83 ± 16.16 HR 78.68 ± 15.69

### 3.3 Efficacy

#### 3.3.1 One‐week follow‐up

##### LVEDV

The pooled effect showed no statistically significant difference between Metoprolol and control group (mean difference [MD] = −2.65, [95% confidence interval [CI] = −7.20 to 1.90], *p* = .25). We observed no statistically significant heterogeneity among studies (*p* = .64, *I* ^2^ = 0%) (Figure S1).

##### LVESV

The pooled effect showed no statistically significant difference between Metoprolol and control group (MD = −4.03, [95% CI = −12.23 to 4.17], *p* = .34). We observed a statistically significant heterogeneity among studies (*p* = .02, *I* ^2^ = 76%), (Figure S2). We did leave‐one‐out test by removing,^12^ and the heterogeneity was solved (*p* = .53, *I* ^2^ = 0%), and the pooled effect showed a statistically significant association between Metoprolol and decreased LVESV compared with the control group (MD = −8.06, [95% CI = −13.40 to −2.72], *p* = .003).

##### LV mass

The pooled effect showed no statistically significant difference between Metoprolol and control group (MD = −1.78, [95% CI = −4.62 to 1.06], *p* = .22). We observed no statistically significant heterogeneity among studies (*p* = .34, *I* ^2^ = 7%) (Figure S3).

##### Infarcted myocardium (%)

The pooled effect showed a statistically significant association between Metoprolol and decreased Infarcted myocardium compared with controls (MD = −3.21, [95% CI = −5.24 to −1.18], *p* = .002). We observed no statistically significant heterogeneity (*p* = .51, *I* ^2^ = 0%) (Figure S4).

##### LVEF (%)

The pooled effect showed no statistically significant difference between Metoprolol and control group (MD = 1.61, [95% CI = −1.08 to 4.29], *p* = .24). We observed a statistically significant heterogeneity among studies (*p* = .009, *I* ^2^ = 79%) (Figure S5). We did leave‐one‐out test by removing,^12^ and the heterogeneity was solved (*p* = .78, *I* ^2^ = 0%), and the pooled effect showed a statistically significant association between Metoprolol and increased LVEF compared with the control group (MD = 2.98, [95% CI = 1.26–4.69], *p* = .0007).

#### 3.3.2 Six‐month follow‐up

##### LVEDV

The pooled effect showed a statistically significant association between Metoprolol and decreased LVEDV compared with the control group (MD = −5.12, [95% CI = −9.18 to −1.05], *p* = .01). We observed no statistically significant heterogeneity among studies (*p* = .58, *I* ^2^ = 0%) (Figure S6).

##### LVESV

The pooled effect showed a statistically significant association between Metoprolol and decreased LVESV compared with the control group (MD = −8.39, [95% CI = −15.8 to −1.70], *p* = .01). We observed a statistically significant heterogeneity among studies (*p* = .009, *I* ^2^ = 67%) (Figure S7). We did leave‐one‐out test by removing,^12^ and the heterogeneity was solved (*p* = .89, *I* ^2^ = 0%), and the pooled effect showed a statistically significant association between Metoprolol and decreased LVESV compared with the control group (MD = −11.13, [95% CI = −15.69 to −6.57], *p* < .00001).

##### LV mass

The pooled effect showed no statistically significant difference between Metoprolol and control group (MD = −1.50, [95% CI = −3.85 to 0.85], *p* = .21). We observed no statistically significant heterogeneity among studies (*p* = .77, *I* ^2^ = 0%) (Figure 8).

#### 3.3.3 Infarcted myocardium (g)

The pooled effect showed a statistically significant association between Metoprolol and decreased infarcted myocardium compared with controls (MD = −3.84, [95% CI = −5.75 to −1.93], *p* < .0001). We observed no statistically significant heterogeneity (*p* = .19, *I* ^2^ = 37%) (Figure S9).

#### 3.3.4 Infarcted myocardium (%)

The pooled effect showed a statistically significant association between Metoprolol and decreased percentage of infarcted myocardium compared with controls (MD = −2.99, [95% CI = −4.27 to −1.70], *p* < .00001). We observed no statistically significant heterogeneity (*p* = .76, *I* ^2^ = 0%) (Figure S10).

#### 3.3.5 LVEF (%)

The pooled effect showed a statistically significant association between Metoprolol and increased LVEF (%) compared with controls (MD = 2.73, [95% CI = 0.71–4.75], *p* = .008). We observed a statistically significant heterogeneity among studies (*p* = .005, *I* ^2^ = 70%) (Figure S11). We did leave‐one‐out test by removing,^12^ and the heterogeneity was solved (*p* = .91, *I* ^2^ = 0%), and the pooled effect showed a statistically significant association between Metoprolol and increased LVEF compared with the control group (MD = 3.57, [95% CI = 2.22–4.92], *p* < .00001).

### 3.4 Adverse events

#### 3.4.1 MACE

The pooled effect showed a statistically significant association between Metoprolol and decreased MACE compared with controls (risk ratio [RR] = 0.84, [95% CI = 0.46–1.53], *p* < .0001). We observed no statistically significant heterogeneity among studies (*p* = .39, *I* ^2^ = 3%) (Figure S12).

#### 3.4.2 Death

The pooled effect showed no statistically significant difference between Metoprolol and controls (RR = 0.58, [95% CI = 0.45–0.76], *p* = .57). We observed no statistically significant heterogeneity among studies (*p* = .83, *I* ^2^ = 0%) (Figure S13).

#### 3.4.3 Heart failure admission

The pooled effect showed a statistically significant association between Metoprolol and decreased Heart failure admission compared with controls (RR = 0.35, [95% CI = 0.18–0.67], *p* = .002). We observed no statistically significant heterogeneity among studies (*p* = .97, *I* ^2^ = 0%) (Figure S13).

#### 3.4.4 Reinfarction

The pooled effect showed a statistically significant association between Metoprolol and decreased reinfarction compared with controls (RR = 0.33, [95% CI = 0.12 to 0.90], *p* = .03). We observed no statistically significant heterogeneity among studies (*p* = .11, *I* ^2^ = 57%) (Figure S13).

#### 3.4.5 Malignant ventricular arrhythmia

The pooled effect showed a statistically significant association between Metoprolol and decreased malignant ventricular arrhythmia compared with controls (RR = 0.49, [95% CI = 0.29–0.85], *p* = .01). We observed no statistically significant heterogeneity among studies (*p* = .99, *I* ^2^ = 0%) (Figure S13).

## 4 DISCUSSION

The β1‐adrenergic‐receptor antagonist such as metoprolol is potentially beneficial in patients with acute myocardial infarction (MI) as they act mainly on cardiomyocytes and decrease the oxygen demand of the myocardium by reducing heart rate blood pressure, and contractility. Some experimental studies also demonstrated that they significantly decrease the risk of ventricular fibrillation. Thus, relative risk reduction in sudden cardiac death has been an overall effect of β1‐adrenergic‐receptor antagonists, demonstrated by some clinical trials.^13^

There is evolving evidence that β1‐adrenergic‐receptor antagonists effectively reduce the infarct size in acute MI patients if given before pPCI and the reduction in mortality. The mechanism behind reducing infarct size with selective β1‐adrenergic agonists such as Metoprolol is somewhat unclear. However, it is most likely attributed to decreasing the inflammation by impairing neutrophil recruitment and neutrophil‐platelet interaction, thus preventing microvascular obstruction.^2^

Here, we report outcomes from a patient‐pooled metanalysis comparing patients with STEMI undergoing pPCI receiving early intravenous β1‐blocker therapy with the control group who did not receive the therapy. We are also comparing the safety of beta‐blocker therapy before pPCI. Our results show that the use of Metoprolol before pPCI was associated with a reduction in the percentage of infarcted myocardium, decreased LVESV, and increased LVEF % at 1‐week follow‐up. Still, there was no significant difference in LVEDV and LV mass between the two groups. However, at 6‐month follow‐up, there was a significant association between the Metoprolol group and decreased LVEDV, decreased LVESV, reduction in myocardial infarct (% and gram), and increased LVEF%. The study also showed a statistically significant association between the Metoprolol group and Decreased MACE, decreased heart failure admission, decreased re‐infarction, and decreased malignant ventricular arrhythmia.

In 2005, Chen et al.^14^ published a randomized placebo‐controlled trial, also known as the COMMIT trial, done on 45 852 patients with MI not undergoing pPCI to study early intravenous followed by oral Metoprolol. Their study results did not show a significant reduction in the outcomes, which included death, reinfarction, or cardiac arrest when compared with the placebo group; however, there was an increase in cardiogenic shock observed mainly during days 0–1 after admission. Thus, given the substantial risk of cardiogenic shock, especially during the first day or so after admission, they suggested considering beta‐blocker therapy only when the hemodynamic condition after MI has been stabilized. Their study, however, is limited by the aggressive dosing and schedule of Metoprolol and includes patients with frank heart failure or pulmonary congestion.

When primary PCI was being used for reperfusion in STEMI, the effect of β‐blockade was further analyzed in a limited and nonrandomized fashion by CADILLAC and PAMI trials in two post hoc retrospective investigations. Their study suggested that the administration of β‐blockers immediately before reperfusion by pPCI resulted in lower mortality when compared to its initiation after reperfusion or no administration at all. However, the effect of preperfusion β‐blockade on infarct size was reported neither in CADILLAC nor in the PAMI trial.^15^, ^16^

It is well established that infarct size predicts post‐infarction mortality. Therefore, treatment with oral β‐blocker within 24 h of a STEMI is a class‐IA indication. Although experimental studies have reduced infarct size after IV β1‐blockade, their use is still not encouraged. Borja Ibanez et al.^8^ conducted a randomized, controlled parallel‐group, observer‐blinded clinical trial of early prereperfusion metoprolol administration in STEMI to study its effects on cardioprotection during acute MI (METOCARD‐CNIC trial) by comparing the pre‐ versus postreperfusion β‐blocker initiation in STEMI. This study revealed that in anterior STEMI patients undergoing primary PCI, administration of early intravenous metoprolol before reperfusion decreases infarct size and increases LVEF. This trial also showed that early intravenous metoprolol was safe and did not increase the cardiac events' incidence during admission.

Similarly, Vincent Roolvink et al.^12^ published a randomized, double‐blind, placebo‐controlled trial that studied the effect of early IV β‐blocker before pPCI in a general STEMI population (EARLY‐BAMI trial). In contrast to the METOCARD‐CNIC trial, this study concluded that Metoprolol reduced the incidence of malignant arrhythmias in the acute phase and was not associated with an increase in adverse events; however, early intravenous Metoprolol before pPCI was not associated with a reduction in infarct size.

Thus, there is a discrepancy in the efficacy of Metoprolol between the METOCARD‐CNIC trial and the EARLY‐BAMI trial. In the METOCARD‐CNIC trial, CMR revealed a reduction in infarct size at 1 week and 6 months using early intravenous β‐blockade. In contrast, no reduction in infarct size was noted at 1 month in the EARLY‐BAMI trial. This discrepancy can be attributed to the difference in dose of IV Metoprolol used in these studies and the timing of dose administration or most likely resulted from a smaller infarct size in the EARLY‐BAMI trial, making it less likely to show a reduction with early β‐blocker use.^17^

Our study is limited by some RoB in two of the included studies in the analysis.

## 5 IMPLICATIONS OF THE STUDY

The results of this study can be interpreted in clinical practice by adding early intravenous metoprolol before PCI in STEMI to the guidelines, as it revealed its significant effect in reducing infarct size and increasing LVEF%.

## 6 CONCLUSION

Our study showed that early intravenous Metoprolol before pPCI was associated with reducing the percentage of infarcted myocardium, decreased LVESV, and increased LVEF % at 1‐week follow‐up. Still, there was no significant difference in LVEDV and LV mass between the two groups. However, at 6‐month follow‐up, there was a significant association between the Metoprolol group and decreased LVEDV, decreased LVESV, reduction in myocardial infarct (% and gram), and increased LVEF%. The study also showed a statistically significant association between the Metoprolol group and Decreased MACE, decreased heart failure admission, decreased reinfarction, and decreased malignant ventricular arrhythmia. More multicenter randomized clinical trials are needed to support our findings.

## CONFLICTS OF INTEREST

The authors declare no conflicts of interest.

## Supporting information

Supporting information.

## Data Availability

All data were analyzed and uploaded as Supporting Information Material.
