# Supplementary material for: Effect of early metoprolol before PCI in ST‐segment elevation myocardial infarction on infarct size and left ventricular ejection fraction. A systematic review and meta‐analysis of clinical trials
Source: Clin Cardiol. 2022 Aug 30;45(10):1011–28. doi: 10.1002/clc.23894 (PMC9574721; doi:10.1002/clc.23894)

## Supplementary Figure1. *LVEDV* at one week

## *
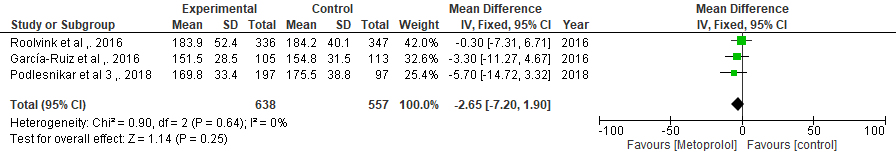
*

## Supplementary Figure2. *LVESV* at one week

##
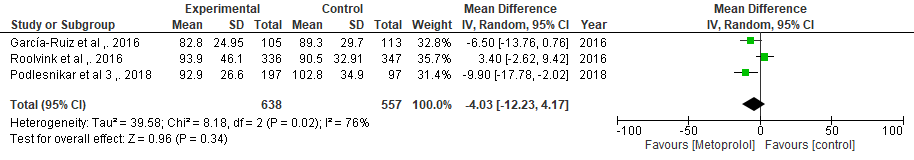


## Supplementary Figure3. LV mass at one week

##
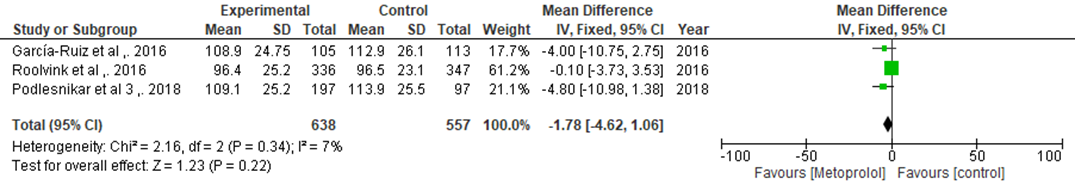


## Supplementary Figure4. Infarcted myocardium (%) at one week

##
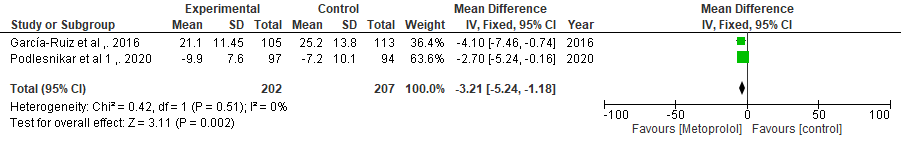


## Supplementary Figure5. LVEF (%) at one week

##
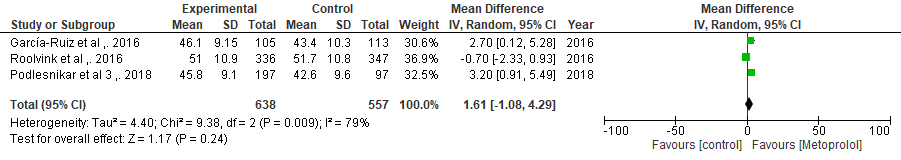


## Supplementary Figure6. *LVEDV* at six months

##
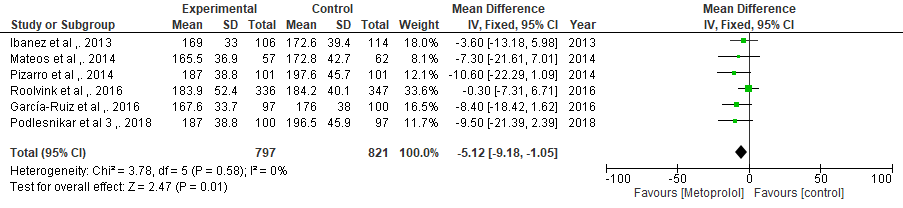


## Supplementary Figure7. *LVESV* at six months

##
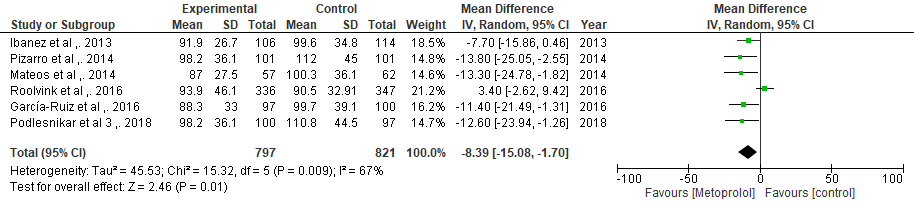


## Supplementary Figure8. *LV mass* at six months

##
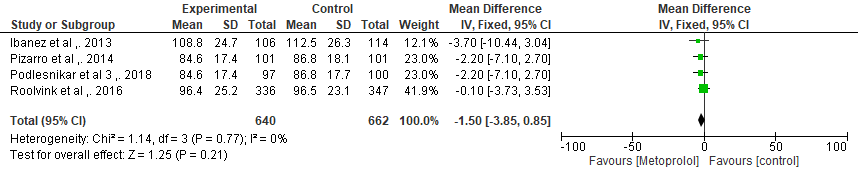


## Supplementary Figure9. Infarcted myocardium (g) at six months

##
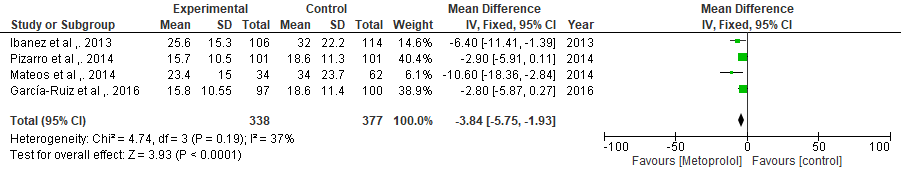


## Supplementary Figure10. Infarcted myocardium (%) at six months

##
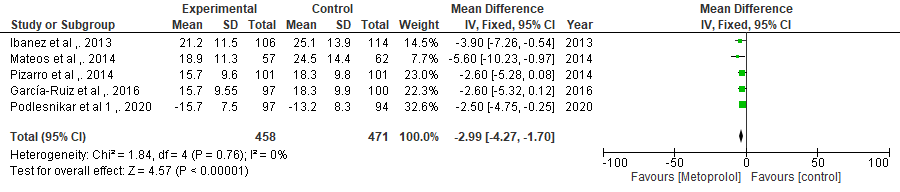


## Supplementary Figure11. LVEF (%) at six months

##
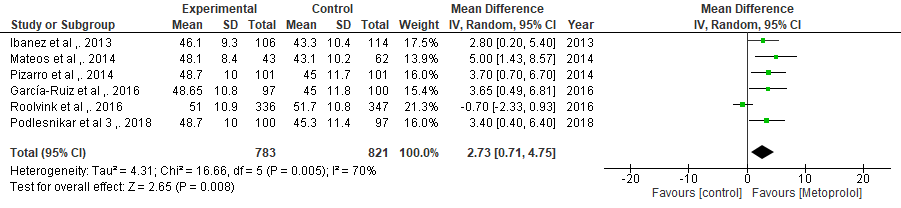


## Supplementary Figure12. MACE

##
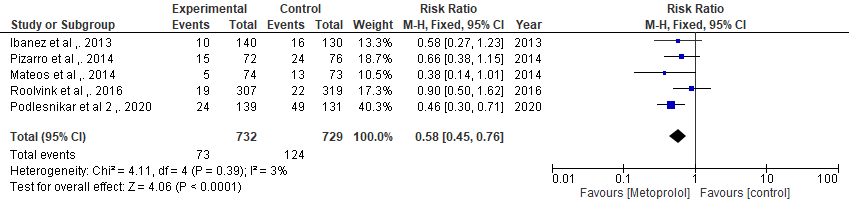


## Supplementary Figure13. All other adverse events

##
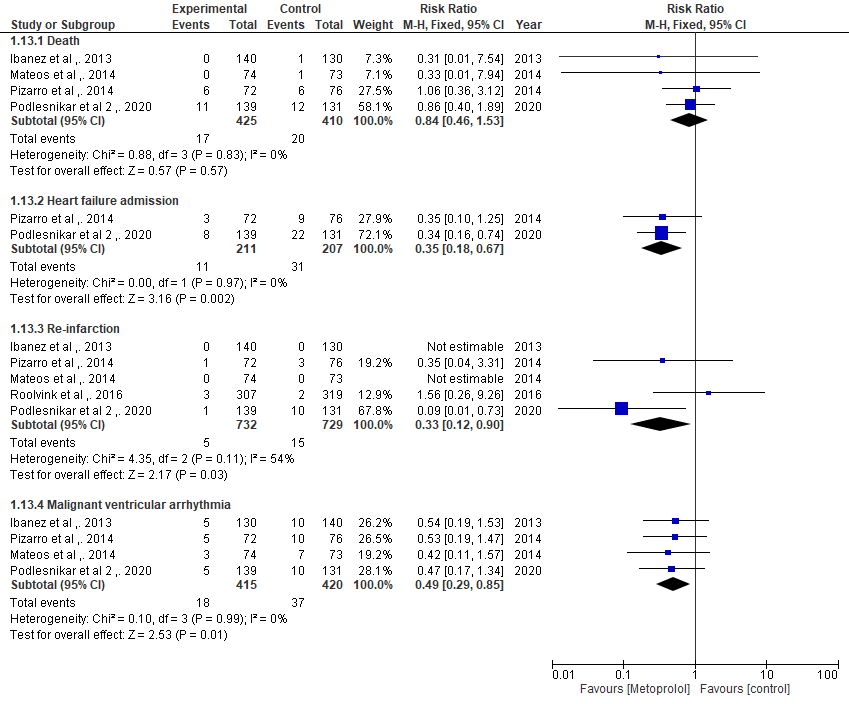

Supplement: Supplementary file 1 — Supporting information. [file CLC-45-1011-s001.docx]
